# How Safe is Eptifibatide during Urgent Carotid Artery Stenting?

**DOI:** 10.3389/fneur.2013.00004

**Published:** 2013-02-12

**Authors:** Hesham Allam, Nirav Vora, Randall C. Edgell, R. Charles Callison, Yasir Al-Khalili, Michelle Storkan, Amer Alshekhlee

**Affiliations:** ^1^Department of Neurology and Psychiatry, Souers Stroke Institute, St. Louis UniversitySt. Louis, MO, USA; ^2^Department of Neurology, University of TexasHouston, TX, USA; ^3^SSM Neuroscience Institutes, DePaul Health CenterSt. Louis, MO, USA; ^4^St. George’s University School of Medicine, St. George’s UniversityGrenada, West Indies

**Keywords:** eptifibatide, carotid stenting, interventional, intracerebral hemorrhage

## Abstract

**Background:** Glycoprotein IIB/IIIA inhibitors are occasionally utilized during carotid artery stenting (CAS) in the presence or absence of a visualized intra-operative thrombus.

**Objective:** We assess the hemorrhagic and clinical outcomes associated with the use of eptifibatide during CAS.

**Methods:** A retrospective analysis of prospectively collected data on patients with the diagnosis of carotid artery stenosis underwent CAS in a single center. We identified those who received intravenous eptifibatide intra-operatively and compared to the rest of the cohort. Hemorrhagic outcomes included intracerebral hemorrhage (ICH) or groin hematoma that occurred during the hospital stay.

**Results:** In this analysis, 81 patients had CAS during a 3-year span; 16 of those had received 15 mg of intravenous eptifibatide intra-operatively. The mean age of the treated and untreated patients was similar (65.6 ± 13.4 versus 65.4 ± 10.2; *P* = 0.13). One patient (1.2%) in this series had ICH in the perioperative period that occurred in the non-eptifibatide group. Five patients (6.2%) in this series had groin hematoma; only one in the non-eptifibatide group required surgical repair. No mortality was reported and clinical outcomes including discharge modified Rankin scale, NIH stroke scale, as well as discharge destination were similar in both groups. A stratified analysis among those who underwent an urgent CAS showed no significant differences in the risks of hemorrhages or any clinical outcome (*P* > 0.05).

**Conclusion:** The use of eptifibatide during CAS is safe. The risk of any hemorrhagic complication is rare in this series; however, a prospective study to validate this observation will be helpful.

## Introduction

In-stent thrombosis is a potential problem associated with carotid artery stenting (CAS); a process attributed to intimal injury during percutaneous arterial interventions resulting in platelet activation and thrombus formation (Gawaz et al., 1996). Some of these processes occur intra-operatively and can be visualized angiographically. Glycoprotein (GP) IIb/IIIa antagonists are a group of drugs that are employed during percutaneous interventional procedures; they work by binding to the GP IIb/IIIa receptors with subsequent inhibition of platelet aggregation and thrombus formation. The GP IIb/IIIa inhibitors may be helpful agents in decreasing the rate of periprocedural ischemic events associated with CAS (Kapadia et al., 2001; Chaturvedi and Yadav, 2006). Eptifibatide is a cyclic peptide derivative that reversibly binds GP IIb/IIIa (Braunwald, 2011). Unlike other agents, intravenous eptifibatide infusion produces high levels of unbound drug. However, its short plasma half-life of approximately 2.5 h allows for rapid clearance and hence, the reversal of platelet inhibition when administration is discontinued. Platelet aggregation has been shown to return to normal in approximately 2–4 h, with the bleeding time normalizing within 1 h (Schror and Weber, 2003).

Because of the transient platelet inhibition, few reports suggested an increased rate of intracerebral hemorrhage (ICH) associated with the use of GP IIb/IIIa antagonists (Chaturvedi and Yadav, 2006). In this series, we assess the incidence of hemorrhagic complications associated with the use of eptifibatide during CAS. In addition, we assess functional outcome, mortality rate, and length of hospital stay.

## Materials and Methods

Approval from the Institutional Review Board was given for our prospective database and an exempt was given to this study. We reviewed all CAS procedures in our database between July 1, 2009 and June 30, 2011; and a cohort of consecutive patients who underwent CAS was selected for analysis. Procedure records were examined including the physician’s procedure report, nursing notes, hospital progress notes, and discharge summaries. We excluded patients if carotid revascularization was emergently needed due to ischemic symptoms occurring within 8 h from onset including those who underwent mechanical thrombectomy. Patients were grouped according to the need for intra-operative administration of intravenous eptifibatide. This need was determined by the primary operator whether for therapeutic or preventative purposes. In addition to patient demographics and vascular risk factors, we collected information on the symptomatic status, preoperative modified Rankin Score (mRS), preoperative NIH stroke Scale (NIHSS), severity of the carotid stenosis, and whether patients were pre-treated with clopidogrel.

### Institution protocol for CAS

Any patient undergoing CAS must meet the center for Medicare and Medicaid services eligibility criteria for high risk symptomatic carotid disease or have high-grade asymptomatic stenosis in the setting of high-risk clinical features (Krajcer, 2005). Two groups of patients were included in this analysis; first, those who were electively admitted but had a qualifying event of stroke or TIA in the prior 3 months, or high risk severe asymptomatic stenosis. The second group included patients admitted for a diagnosis of TIAs or mild stroke and urgent CAS was needed due to fluctuating neurological symptoms or recurrent TIAs (but after 8 h from symptoms onset). Patients with a large core infarction (more than 20% of the territory supplied by the middle cerebral artery) based on brain MRI and computerized tomography scans are excluded from immediate CAS, i.e., revascularization during the first 4 weeks from the onset of the ischemic strokes. The severity of the stenosis based on carotid angiography was measured using the North American Symptomatic Endarterectomy Trial criteria methodology (North American Symptomatic Carotid Endarterectomy Trial Collaborators, 1991). In the pre-operative period, patients received dual antiplatelet therapy with Aspirin (325 mg daily) and clopidogrel (75 mg daily) for at least 3 days. If the patient had not been on clopidogrel for at least 3 days, a 300 mg bolus was administered at least 6 h prior to performing the CAS. CAS procedures were performed in a bi-plane angiography suite under conscious sedation; with vascular access through the femoral artery. Once the femoral sheath was in place, all patients received a bolus of intravenous heparin to achieve activated clotting time of 250–350 s (Saw et al., 2006). Distal embolic protection device was utilized in all cases; this was followed by a mono-rail stent assisted balloon angioplasty, and stent deployment. If angiographic platelet aggregation or thrombus formation is visualized, a bolus of 15 mg of eptifibatide is administered intravenously. Occasionally, eptifibatide was used preventatively if clopidogrel was not given pre-operatively. No patients were placed on continuous intravenous eptifibatide drips. At the conclusion of the CAS procedure, a vascular closure device, or manual compression of the femoral artery was used based on operator preference, the presence of vascular disease in the femoral angiography, or a puncture site at the femoral bifurcation. All patients after CAS were admitted to the neurointensive care unit for close hemodynamic and neurological monitoring and management. Critical care management may last for 24–48 h or longer if symptomatic management of the blood pressure is needed or clinical event had occurred.

### Statistical analysis and outcomes

Chi-square and Fisher’s exact tests were used to compare categorical proportions; and *t*-test and Wilcoxon rank sum test were used to compare the mean and median of continuous variables. The primary outcome was ICH that was identified during the hospital stay or after patients were discharged from the hospital. ICH was identified by conventional or DYNA computerized tomography scans, and confirmed by MRI when appropriate. All groin hematomas were reported including the clinically significant hematoma (hematoma that needed a surgical repair or blood transfusion). In addition, we identified functional outcomes based on the discharge mRS as well as the NIHSS. Lastly, we assessed the length of stay, discharge locations, and hospital mortality rates. A stratified analysis was performed for those who were hospitalized for acute stroke or TIA and in whom CAS was performed during the same hospital stay.

## Results

From the initial sample of 87 patients, 6 were excluded because was performed as part of an intervention for ongoing acute ischemic stroke, including those who underwent mechanical thrombectomy. Eighty one patients with the mean age of 65.5 were included in this analysis; 16 were treated with eptifibatide during CAS. The mean age of the treated and untreated population was similar (65.6 ± 13.4 versus 65.4 ± 10.2; *P* = 0.13). Males were predominant in both groups (75 versus 64.1%; *P* = 0.4). Eight patients (9.8%) were asymptomatic with the remainder of the sample having had TIA, stroke, or amaurosis fugax, 22.2, 62.9, and 3.7%, respectively. The proportion of symptomatic patients did not differ in each group (Table 1). Fourteen of 16 (87.5%) patients in the eptifibatide group and 42/65 (64.6%) in the non-eptifibatide group had acute stroke or TIA upon presentation and underwent CAS during the same hospital stay (*P* = 0.07). Vascular risk factors of diabetes, hypertension, coronary, and peripheral arterial disease, hyperlipidemia, history of TIA or stroke, and smoking were similar in both groups (*P* > 0.05). Similarly, the mean mRS (1.18 ± 1.10 versus 0.84 ± 0.91) upon initial hospitalization as well as the stroke severity (NIHSS: 1.75 ± 2.26 versus 2.06 ± 2.70) were similar in both groups (*P* > 0.05).

**Table 1:** **Cohort demographics**.

	CAS + eptifibatide *N* = 16	CAS − eptifibatide *N* = 65	*P* value
Age (mean ± SD)	65.6 ± 13.4	65.4 ± 10.2	0.13
**GENDER *n (%)***			
Female	4 (25.0)	23 (35.9)	0.40
Male	12 (75.0)	41 (64.1)	
**SYMPTOMATIC STATUS *n (%)***			
Asymptomatic	2 (13.3)	6 (9.23)	0.47
TIA	3 (20.0)	15 (23.1)	
Stroke	10 (66.7)	41 (63.1)	
Amourosis Fugax	0	3 (4.6)	
TIA or stroke upon initial presentation* *n* (%)	14 (87.5)	42 (64.6)	0.07
**VASCULAR RISK FACTORS *n (%)***			
Diabetes	5 (31.2)	22 (33.8)	0.84
Hypertension	14 (87.5)	58 (89.2)	0.84
Coronary artery disease	10 (62.5)	27 (41.5)	0.13
Hyperlipidemia	11 (68.7)	45 (69.2)	0.97
Smoking	6 (37.5)	19 (29.2)	0.52
Peripheral artery disease	1 (14.3)	8 (27.6)	0.46
TIA or stroke	8 (53.3)	32 (50.8)	0.85
**PRE-STENT (MEAN ± SD)**			
mRS	1.18 ± 1.10	0.84 ± 0.90	0.26
NIHSS	1.75 ± 2.26	2.06 ± 2.70	0.86
Stenosis severity rate	82.62 ± 25.6	84.9 ± 14.8	0.72
Pre-stent clopidogrel *n* (%)	14 (87.5)	58 (89.2)	0.84

*This group had acute neurological changes of either TIA or small stroke and carotid artery stenting (CAS) performed during the same hospital stay.

In this cohort, one patient in the non-eptifibatide group (1.2%) had ICH following CAS that was attributed to reperfusion syndrome (Wu et al., 2012). Five patients developed groin hematomas in this cohort, only one was deemed as clinically significant occurred in the non-eptifibatide group and required vascular repair. Among the eptifibatide group, one patient (6.2%) had a groin hematoma that did not require blood transfusion or surgical intervention. Functional status measured by discharge mRS was similar in both groups (0.54 ± 0.74 versus after 0.88 ± 0.98). The length of hospital stay was similar in both groups (*P* = 0.44). In this cohort, no mortality was reported and 56 of 81 patients (69.1%) discharged to home; and 25 (30.8%) patients discharged to rehabilitation centers. Angiographically, this cohort demonstrated severe carotid disease with the mean stenosis rate of 84.1%; which was similar in both groups (Table 1). Postoperatively, the stenosis rate was 13.7%; which again was similar in both groups (Table 2). All patients were pre-treated with aspirin before CAS; 72 patients (88.9%) were given clopidogrel prior to the procedure. When the sample was stratified by those who underwent urgent CAS during the same hospital stay, similar results were found (Table 2). No ICH or mortality was reported but four groin hematomas (one in the eptifibatide treated and four in the non-eptifibatide groups).

**Table 2:** **Outcomes associated with carotid artery stenting (CAS) in association with the use of eptifibatide among all patients, and a subgroup of patients with acute stroke or transient ischemic attacks followed by urgent CAS**.

	All patients with CAS			Patients with urgent CAS		
	CAS + eptifibatide *N* = 16	CAS − eptifibatide *N* = 65	*P* value	CAS + eptifibatide *N* = 14	CAS − eptifibatide *N* = 43	*P* value
Intracerebral hemorrhage *n* (%)	0	1 (1.5)	0.61	0	0	–
Any groin hematoma *n* (%)*	1 (6.2)	4 (6.1)	0.98	1 (7.1)	3 (7.1)	0.99
Post-stent stenosis rate (mean ± SD)	7.66 ± 9.42	14.0 ± 18.8	0.34	8.21 ± 9.52	14.5 ± 20.3	0.51
Discharge mRS (mean ± SD)	0.54 ± 0.74	0.88 ± 0.98	0.21	0.61 ± 0.76	1.07 ± 1.27	0.18
Discharge NIHSS (mean ± SD)	1.20 ± 1.65	1.83 ± 2.79	0.51	1.38 ± 1.70	2.46 ± 3.25	0.37
Length of stay – days (mean ± SD)	3.66 ± 3.1	4.7 ± 4.9	0.66	4.1 ± 3.1	6.27 ± 5.54	0.20
**DISCHARGE LOCATION *n (%)***						
Home	11 (64.7)	45 (69.2)	0.54	8 (61.5)	25 (60.9)	0.97
Rehabilitation center	5 (31.2)	20 (31.2)		5 (38.5)	16 (39.1)	

*Any groin hematoma refers to all hematoma that are clinically and non-clinically significant.

## Discussion

Intimal injury during CAS may lead to collagen exposure with subsequent activation of procoagulant factors with the end result of a platelet-rich thrombus formation. In some instances, thrombus formation may cause occlusion of a cerebral blood vessel with subsequent ischemic stroke. Interventionalists utilize GP IIb/IIIa inhibition during CAS either therapeutically to treat acute in-stent thrombosis or preventatively to reduce the risk of periprocedural thrombus formation. Few reports suggested that the use of GP IIb/IIIa inhibitors alone or in combination with intra-arterial thrombolysis may lead to successful revascularization of an acutely thrombosed stent during CAS (Tong et al., 2000; Steiner-Boker et al., 2004). In a retrospective review of 254 CAS procedures, Green et al. identified two patients with witnessed thromboembolic events occurred intra-operatively. In both patients, the thrombotic events occurred during the initial passage of the filter wire through the proximal lesion. Nevertheless, both patients were successfully treated with intra-arterial urokinase and intravenous GP IIb/IIIa inhibitor (abciximab; Green et al., 2005). Adjunctive use of GP IIb/IIIa inhibitors during interventional procedures has been shown to decrease the risk of periprocedural ischemic events (Qureshi et al., 2004; Dumont et al., 2012). Most of these data were abstracted from the cardiology literature, which has shown that the adjunctive use of GP IIb/IIIa inhibitors in the setting of percutaneous coronary intervention significantly reduces the rates of 30-day mortality and myocardial Infarction, as well as reduces the need for repeat revascularization procedures (Labinaz et al., 2007; Winchester et al., 2011). These beneficial effects were achieved at an increased risk of thrombocytopenia and minor bleeding.

In our study, we found a low risk of ICH and groin hematoma associated with the use of eptifibatide during CAS. The overall rate of ICH in our cohort (1.2%) is compatible with previous reports which ranged between 0.36 and 4.1% (Cheung et al., 2003; Moulakakis et al., 2009). None of the 14 patients with acute stroke and treated with eptifibatide during CAS had ICH. There has been contradicting safety data regarding the use of GP IIb/IIIa inhibitors during CAS (Qureshi et al., 2002; Chan et al., 2005; Kramer et al., 2007; Zahn et al., 2007) however, most of these studies were focused on abciximab during CAS. Kapadia et al. evaluated 151 patients with CAS, 128 of those had been prophylactically treated with abciximab while the rest of cohort was treated with intravenous heparin. At 30-days, the thromboembolic rates were significantly less in the abciximab group (1.6 versus 8%) with one patient developed ICH in the abciximab group (Kapadia et al., 2001). The authors suggested a relative safety of abciximab during CAS. On the contrary, Wholey et al. shown higher rates of thromboembolic events (6 versus 2.4%) as well as hemorrhages in abciximab group compared to intravenous heparin. Two of four neurologically related deaths in the abciximab group were due to large ICH compared to no hemorrhages in the heparin group (Wholey et al., 2003). These observations led the authors to conclude relative risks associated with the use of GP IIb/IIIa inhibitors during CAS. A recent change in focus to the use of eptifibatide during cardiac interventional procedures has been noted by neurointerventionalists (Mahmoudi et al., 2011). Eptifibatide has a shorter life span compared to abciximab and hence, may have a better safety profile. The safety and efficacy of eptifibatide was assessed in a small sample of CAS patients treated with high-dose eptifibatide administered as intravenous bolus followed by continuous infusion over 20–24 h post-operatively (Qureshi et al., 2004; Dumont et al., 2012). Again the authors suggested eptifibatide was safe to use during CAS. Besides the absent risk of ICH in our eptifibatide group, no mortality was reported despite the fact that more than half of our patients had CAS performed urgently.

This study is limited by the small sample of those who were treated with eptifibatide during CAS, thus may have led to a selection bias. In addition, the use of eptifibatide in our series was not uniform, i.e., eptifibatide was given for therapeutic and preventative purposes. Regardless, this study suggests the relative safety of eptifibatide use during CAS when performed electively or urgently. Risk-benefit assessment in large prospective studies or national registry would be useful.

## Conflict of Interest Statement

The authors declare that the research was conducted in the absence of any commercial or financial relationships that could be construed as a potential conflict of interest.
